# Optimization Analysis for Pavement Construction Integrated Optical Fiber Sensors Based on DEM-FDM Coupled Method

**DOI:** 10.3390/ma19061221

**Published:** 2026-03-19

**Authors:** Peixin Tian, Min Xiao, Yaoting Zhu, Xihai Yang, Yongwei Li, Xunhao Ding, Tao Ma

**Affiliations:** 1 School of Transportation, Southeast University, 2 Sipailou, Nanjing 210096, China; 220233446@seu.edu.cn (P.T.); matao@seu.edu.cn (T.M.); 2 Jiangxi Communications Investment Group Co., Ltd., No. 367, Chaoyangzhou Middle Road, Nanchang 330025, China; xiaom123555@163.com (M.X.); yangxihai12345@163.com (X.Y.); liyw123876@163.com (Y.L.); 3 Jiangxi Communications Investment Maintenance Technology Group Co., Ltd., No. 809, Jinsan Avenue, Xiaolan Economic Development Zone, Nanchang 330052, China; yaoting_z2020@163.com

**Keywords:** road construction, discrete element method-finite difference method coupling, distributed fiber-optic sensing, performance of optical fiber sensors

## Abstract

Today, distributed optical fiber sensors are widely used in structural health monitoring due to their high sensitivity and long-distance applicability. However, when embedded in pavement structures, distributed optical fiber sensors are always installed in a slotted buried fashion, which not only affects current pavement durability but also reduces pavement construction efficiency. In order to design clear requirements of in situ-embedded distributed optical fiber sensors for pavement construction, this study analyzes the micro-mechanical behavior of optical cables under the ultimate pavement compaction state based on a coupled DEM-FDM approach. According to the study results, it is found that when the pavement subbase was compacted, the maximum contact force of 13.2 mm aggregates in the *Z*-direction exceeds 150 N, which is the main resistance of the external load during pavement construction. The tight-buffered optical cable without reinforcement element and armored layer cannot withstand the vibration load. The inclusion of GFRP strengthening components and an armored layer decreased maximum stress by 38.2% (*X*), 30.6% (*Y*), and 30.9% (*Z*), as well as displacement by 64.6% (*X*), 45.5% (*Y*), and 66.7% (*Z*). Additionally, the thickness of the outer sheath enhanced the ability to withstand tension but not compression. The increase in the thickness of the armored layer can improve the ability to withstand tension and compression.

## 1. Introduction

In recent years, increasing attention has been given to the health monitoring of transportation infrastructure. Pavement monitoring systems have increasingly evolved toward becoming automated, continuous, and data-driven. Unpaved pavement are only equipped with dynamic monitoring systems, while paved pavement are equipped with both static and dynamic monitoring systems, covering asphalt and concrete pavements. Monitoring technologies include inertial profilometers and laser systems for the detection of roughness and surface defects, ground-penetrating radar (GPR) for the assessment of layer thickness and moisture conditions, and automated defect detection technologies based on digital imaging [1]. An intelligent pavement defect detection system was constructed by Guerrieri et al. [2]. By integrating video capture devices with the YOLOv3 object detection algorithm, it enables real-time identification of various asphalt pavement defects on urban roads crossing tram lines. Monitoring pavements is an effective method for evaluating road usage. Optical fiber sensing technology is characterized by high sensitivity, immunity to electromagnetic interference, corrosion resistance, and the capability for long-distance monitoring. Optical fiber sensing technology has been widely utilized for the health monitoring of pavements [3,4].

According to its technical characteristics, optical fiber sensing technology can be categorized into quasi-distributed Fiber Bragg Grating (FBG) sensors and fully Distributed Optical Fiber Sensing Systems (DOFS) [5,6]. The sensing principle of FBG involves using Bragg gratings in optical fibers to reflect light at a specific wavelength, while transmitting light at other wavelengths. The changes that occur in the grating period and refractive index when FBG is subjected to external factors such as strain or temperature lead to alterations in the Bragg wavelength. The alterations can be measured to determine the applied strain or temperature change, rendering FBG suitable for monitoring temperature and strain in road structures [7]. DOFS utilizes the inherent properties of optical fibers for continuous distributed measurements along the fiber. According to their respective scattering principles, DOFS can be categorized into Rayleigh scattering, Raman scattering, and Brillouin scattering. Rayleigh scattering can be primarily characterized by the elastic scattering of light. The frequency of Rayleigh scattering remains constant. In Rayleigh scattering, the wavelength and frequency of scattered photons are identical to those of incident photons. Optical Time-Domain Reflectometry (OTDR) and Optical Frequency-Domain Reflectometry (OFDR), which are based on Rayleigh scattering, can be utilized to monitor vibrations caused by external disturbances and fiber tension along the optical fiber [8]. Raman and Brillouin scattering are inelastic scatterings that result in a frequency shift in the scattered light. Raman scattering, caused by molecular vibrations, involves the interaction of incident light with optical phonons [9]. Raman Optical Time-Domain Reflectometry (ROTDR) can be employed for temperature monitoring [10]. Brillouin scattering involves inelastic scattering due to material density fluctuations as light propagates through the fiber, resulting in frequency shifts in two directions. The shifts are influenced by factors such as fiber sound velocity, refractive index, composition, temperature, and strain [11]. Brillouin Optical Time-Domain Analysis (BOTDA) and Brillouin Optical Time-Domain Reflectometry (BOTDR) monitor strain and temperature changes along the optical fiber by measuring the frequency changes in the scattered light [12].

Since uncoated optical fibers are extremely fragile, structural failures could occur when they are subjected to road construction loads and traffic loading. Therefore, distributed optical fiber sensors were required to be encapsulated to withstand potential external loading, which ensures the survivability of the sensors [13]. Due to the strain transfer mechanism, a portion of energy was absorbed by the encapsulation layer, which generated a strain transfer coefficient between the strain measured within the structure and the actual structural strain [14]. Before deploying optical fiber sensors, the strain calibration coefficients are typically determined through dedicated calibration tests to facilitate subsequent corrections to the measurement results. Chen et al. [15] proposed a calibration method using standard beams and mature electric resistance strain gauges (ESG) of equal strength to calibrate the accuracy of FBG and OFDR. Furthermore, the encapsulation design, the properties of the host material, and embedding method of optical fiber sensors substantially influence measurement accuracy. Li et al. [16] studied the reliability of FBG sensors through fatigue tests, concluding that the number and size of CFRP encapsulations influence FBG sensor reliability. Majumder et al. [17] measured strain curves using Rayleigh scattering-based optical demodulators with telecommunication optical cables embedded in pavement layers as distributed sensors at the IFSTTAR fatigue carousel in France. The Federal Aviation Administration (FAA) embedded 24 fiber-optic strain gauges using white light polarization interferometry (WLPI) technology bonded with epoxy resin at the National Airport Pavement Test Facility in Atlantic City, New Jersey [18]. Van den Bergh et al. [19] configured FBG sensor systems on the surface of prefabricated asphalt specimens and embedded the sensors in bicycle lanes and grooves, protected with high-density polyethylene (HDPE) coatings. Liu et al. [20] placed fiber-optic sensors in grooves excavated at the top of the subgrade on sections of the Hefei–Dajin Expressway in China, which were compacted with fine soil before constructing the upper cement-stabilized aggregate layer. Zhang et al. [21] embedded sensing cables through vertical boreholes in the soil, backfilled with soil to complete self-consolidation, thereby obtaining multi-field multi-parameter data during ground subsidence. Huang et al. [22] employed metal-based and tight-buffered sensing cables along anchor bolts, which were uniformly coated with epoxy resin. They measured axial strain during pull-out tests by BOTDR, thereby evaluating the anchorage performance of anchor bolts.

For pavement health monitoring, it is essential to embed distributed fiber-optic sensors in asphalt mixtures. Currently, most of the distributed optical fiber sensor laying schemes are reopened or buried by anchoring and drilling after the construction of the pavement. However, in practical pavement monitoring applications, strain measurement errors persist owing to the discrepancies among various installation techniques. Chapeleau et al. [23] conducted full-size field tests by embedding fiber sensors directly on the asphalt structure and covering with asphalt mortar or sealant. The fiber sensors located in the lower grooves were found to have survived the heavy mechanical structure. Liu et al. [24] employed distributed acoustic sensing (DAS) for traffic-flow monitoring and event detection. The results confirmed its stability and engineering viability for vehicle-event identification. However, high temperatures, compaction, and vibration during road construction are significant causes of sensor damage, severely affecting measurement accuracy. Further research is needed to determine whether optical fiber sensors meet the needs of road construction tolerance and ensure the reliability of monitoring.

Numerical simulation provides a complementary route for analyzing the coupling between optical cables and their surrounding media. The numerical simulation mainly utilizes the discrete element method (DEM) and the finite difference method (FDM). The coupled DEM-FDM approach was largely utilized to characterize the interactions between granular materials and continuous media. A comprehensive macroscopic and microscopic analysis of a railway ballast and subgrade system was successfully achieved by Shi et al. [25]. The ballast particles were treated as discrete elements while the subgrade was modeled as a continuous body. In this study, both the discrete particle characteristics of graded crushed stone and the continuous mechanical principles of the DFOS could be simultaneously evaluated by implementing the coupled DEM-FDM. Consequently, the approach was highly conducive to analyzing the complex interactions between the distributed optical fiber sensors and the graded aggregates. Song et al. [26] investigated cone penetration in sand by coupling the discrete element method and the finite difference method. After micro–macro parameter calibration, they elucidated the cone-resistance mechanism and established the relationship between normalized cone resistance and internal friction angle. Dong et al. [27] adopted a DEM–FDM framework to evaluate the accuracy of distributed fiber-optic sensing (DFOS) in asphalt mixtures and developed a strain-transfer model, quantifying the dominant influence of interfaces and sensor packaging on measurement accuracy. Gu et al. [28] analyzed the mechanical coupling between loose granular media and optical cables using DEM and showed that peaks in the contact-force chain govern cable response and measurement distortion.

To investigate the performance change in optical fiber sensor during pavement construction, DEM-FDM (combining the discrete element method with the finite difference method) was utilized for dual-scale coupled numerical simulation. Considering the discrete characteristics of graded aggregates, the discrete element software Particle Flow Program (PFC3D, version 7.0) was used to describe the graded aggregates and finite differential software Fast Lagrangian Analysis of Continuum (FLAC3D, version 7.0) to describe DFOS to establish the coupling model of a distributed fiber sensor embedded in a pavement structure. In order to represent the most unfavorable pavement construction conditions, the distributed fiber sensor was located on the top of the subgrade. Under vibratory compaction of the pavement subbase, the effects of cable structural design form and size on the mechanical performances and the spatial position error of optical cable were analyzed. The results can provide references for pavement construction integrated DOFS sensor deployment.

## 2. Methodology

### 2.1. Dual-Scale Coupled Model Establishment for Optical Cable Embedded in Pavement

Optical fiber is a cylindrical, symmetrical structure comprising a central core and an outer layer. The outer layer can be coated with inner and outer coatings. Optical cable is constructed by using appropriate materials and cable structure to provide containment protection for the optical fiber, shielding it from mechanical and environmental impacts and damage. The optical cable consists of a cable core, strengthening components, and an outer sheath [29,30].

**Figure 1:**
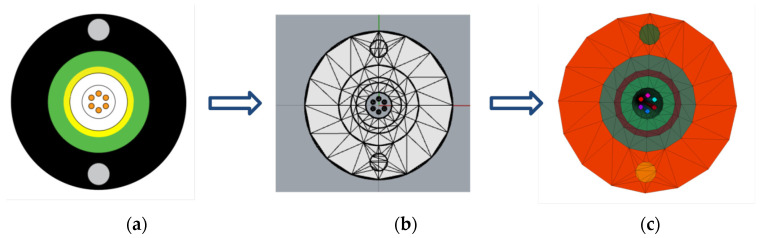
Cable model establishment: (**a**) two-dimensional diagram of optical cable; (**b**) optical cable division grid; (**c**) finite element model of optical cable.

A two-dimensional cross-section of the optical cable was created using the auxiliary design software AutoCAD 2025, as shown in Figure 1a. The DWG file was subsequently imported into the 3D modeling software Rhinoceros (v.7.0 by McNeel & Associates, Seattle, WA, USA). The NURBS plugin in Rhino, with the rotation formation command, was employed to construct a 3D model of a fully triangular curved mesh optical cable with a minimum edge size of 0.1 m, as shown in Figure 1b. The f3grid file, which contains the 3D model of the optical cable exported from Rhino, was imported into FLAC3D. The 3D finite element model of the optical cable is shown in Figure 1c.

The research site was located in Shandong Province, China, on the national expressway G22 that connects Qingdao and Lanzhou, within the segment between the Dong’e boundary and Liaocheng. The segment was connected to the expressway section between Tai’an and the Dong’e boundary on the eastern side and linked to the Handan to Daming Expressway in Hebei Province on the western side. It traversed five administrative districts in Shandong Province along an alignment from east to west. Based on the pavement structure of the segment, a finite element model of the subgrade was developed in FLAC3D. The model measured 1.0 m in length, 0.2 m in width, and 0.4 m in height, with an elastic modulus of 60 MPa and a Poisson’s ratio of 0.40. Three-dimensional hexahedral elements were adopted for the mesh generation. The grid sizes set to 0.05 m and 0.04 m in the horizontal and vertical directions. The Mohr–Coulomb elastoplastic model was selected as the constitutive model for the subgrade soil. Soil density, elastic modulus, Poisson’s ratio, cohesion, and internal friction angle were obtained through laboratory triaxial shear tests and standard compaction tests. Specifically, the cohesion and internal friction angle were determined to be 33.6 kPa and 19°. The other constitutive model parameters of the subgrade are summarized in Table 1.

A discrete element model of graded aggregates was generated using the PFC3D software package within a spatial domain above the subgrade finite element model. The spatial domain measured 1 m in length, 0.2 m in width, and 0.4 m in height. The loose diffusion thickness was controlled at 400 mm so that particles could move vertically. Four representative aggregates, specifically 2.36, 4.75, 9.5, and 13.2 mm, were selected for this investigation. Computed tomography (CT) technology was employed to obtain cross sectional images of aggregate particles. The images were meshed in Simpleware to produce STL files that were imported into PFC3D to generate coarse aggregate clump templates with realistic three-dimensional real morphology. The clump elements were employed to represent graded aggregate composed of pebble elements. Fine aggregates smaller than 2.36 mm exert a negligible effect on the formation and stability of the skeleton structure. Consequently, in the numerical simulation model, asphalt mortar particles are omitted, focusing solely on the graded aggregates. Furthermore, the simulated particle sizes were maintained to be consistent with those of the actual aggregates. The gradation for aggregate skeletons are listed in Table 2.

**Figure 2:**
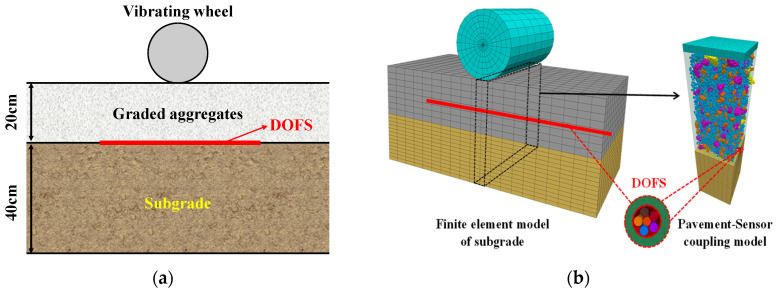
DEM-FDM coupled model establishment: (**a**) Optical cable layout diagram; (**b**) Coupling model for distributed fiber sensor embedded in pavement.

Boundary walls were created around the aggregate skeletons assembly. The distributed fiber sensor was located on the top of the subgrade, as indicated in Figure 2a. A coupling wall was defined at the interface between the finite-element domain and the aggregate skeletons model to enable mechanical interaction between the discrete element aggregate model and the finite element model of embedded cable. The boundary conditions of the coupling model are set so that the bottom of the subgrade is fixed, and horizontal displacements around the perimeter of the subgrade surface are restricted. The parameters of the finite element models for the distributed fiber optical cable and pavement are shown in Table 1.

To investigate the interaction between the embedded optical cable and crushed-stone particles under vibratory compaction, a finite-element model of a loading plate was established on the top of the aggregate skeletons to simulate a stee wheel roller. A loading plate model with dimensions of 1 m in length, 0.2 m in width, and 0.1 m in height was integrated into the coupled system for the purpose of applying compaction loads. A coupling wall was also established at the interface between the loading plate and the aggregate skeletons to ensure accurate load transfer. The movement of the wall was precisely controlled by a servo mechanism and periodically adjusted to reach a new state of force equilibrium under gravity. Consequently, a dual-scale coupled model for an optical cable embedded in pavement was established, as shown in Figure 2b.

### 2.2. Numerical Simulation of Vibration Compaction

In the numerical analysis of vibratory compaction, the roller was simplified as a single vibratory drum. The inertial mass of the steel drum was considered. Meanwhile, the vibratory contribution of the frame was neglected, and its mass was represented as a static vertical load applied at the drum centroid [28]. The compaction force exerted by the roller on the asphalt pavement can be approximated as the vertical component of the eccentric force generated by the roller’s gravity and eccentric block. The repeated impact load during vibratory rolling was equivalent to a constant force acting under optimal conditions of maximum excitation force. The separation of the eccentric force in the vertical direction can be calculated as:

(1) $$F_{e}=M_{d}A_{0}{\left(2\pi f\right)}^{2}$$

where $F_{e}$ is the excitation force; $M_{d}$ is the mass of the vibrating wheel; $A_{0}$ is the nominal amplitude; and f is the excitation frequency.

Vibratory compaction generally consists of three stages: initial compaction, intermediate compaction, and final compaction. In this study, initial compaction was conducted with a double-drum vibratory roller following the paver with one static pass. Intermediate vibration compaction utilized a single-drum vibratory roller with intense vibration and final compaction was performed with a double-drum vibratory roller with one static pass. The vibratory-roller parameters were selected with reference to the XS263J single-drum vibratory roller from Xuzhou Construction Machinery Science and Technology Co., Ltd. (Xuzhou, China), whose primary parameters are shown in Table 3. Before applying the loading plate, the displacements and velocities of all discrete-element particles in the graded aggregates were reset to zero to initialize the state variables. The excitation force was applied to the loading plate, and compaction was terminated once the compaction height reached the prescribed subbase thickness of 20 cm for the national expressway segment.

**Figure 3:**
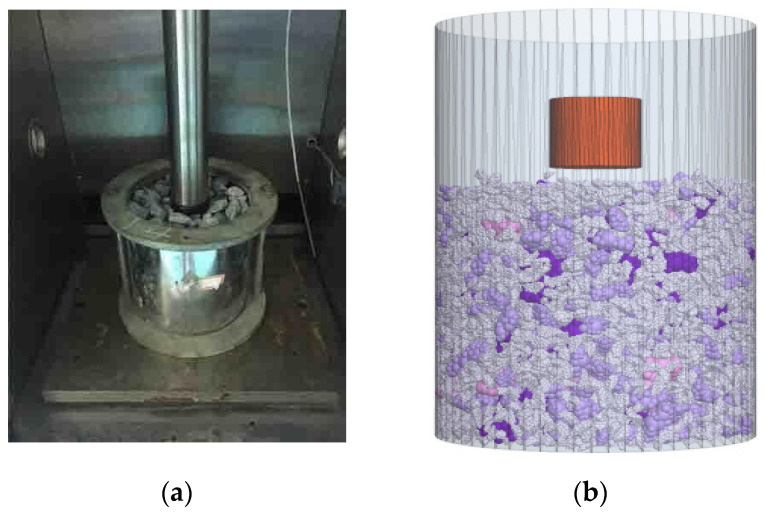
Laboratory uniaxial penetration test and virtual uniaxial penetration test: (**a**) Laboratory uniaxial penetration test; (**b**) Virtual uniaxial penetration test.

In order to make the simulation closer to the practical application situation, it is necessary to calibrate the relevant contact parameters between the aggregates. As shown in Figure 3a, the basalt coarse aggregates were used for penetration test based on Universal Testing Machine (UTM). The penetration test was conducted to determine the microscopic contact parameters governing the interactions among aggregate particles. Consequently, the numerical model could be enabled to more accurately reflect the mechanical transfer characteristics of the actual aggregates. The aggregates were firstly compacted into specimens with diameter of 10 cm and height of 15 cm. The loading head is 50 mm and specimens were penetrated at a constant vertical speed of 1 mm/min. Aggregates with single size of 13.2, 9.5, 4.75 and 2.36 mm were penetrated individually to obtain the contact parameters in the discrete element model.

According to JTG E20-2019 [32], the specimen dimensions were determined as a cylinder with a diameter and height of 100 mm each. Aggregates were batched and placed in a space twice the height of the specimen size. Gravity was applied, causing the aggregate particles to undergo volume scaling and self-weight settlement. A loading plate descending at a constant speed was then applied at the top, gradually lowering it to the preset height of the specimen to compact the aggregates. After removing the loading plate, the model was continuously cycled to fully adjust particle positions and eliminate unbalanced forces. Specimens of skeletons with single particle size were resulted in the model, including 13.2, 9.5, 4.75 and 2.36 mm aggregates. A cylindrical indenter with a diameter of 28.5 mm was placed at the center of top surface of the gradation skeleton models. The indenter moved downward at a constant rate 1 mm/min, as shown in Figure 3b.

The aggregates for this study consist of irregularly shaped blocks that interlock, resulting in little particle rotation. Therefore, the contact model of the aggregates is Linear Parallel Bond Contact Model. The Linear Parallel Bond Contact Model enhances the linear model by incorporating additional internal moments between contacting particles, which increase with particle rotation, and the model reduces particle rotation during simulation. The Linear Parallel Bond Model contact model resembles the linear model in which normal and tangential stiffness follow linear contact models. Internal moments increase linearly with accumulated relative rotation at contact points. The accumulation equals the product of tangential stiffness and effective contact radius in the linear model. The constant term between the increment of internal moments and the increment of relative rotation at contact points was defined as torsional stiffness.

The formula of normal stiffness and tangential stiffness was calculated as:

(2) $$\left\{\begin{array}{c} k_{n}=\frac{k_{n}^{A}k_{n}^{B}}{k_{n}^{A}+k_{n}^{B}}\\ k_{s}=\frac{k_{s}^{A}k_{s}^{B}}{k_{s}^{A}+k_{s}^{B}} \end{array}\right.$$

where $k_{n}$ is the total normal contact stiffness; $k_{s}$ is the total tangential contact stiffness; $k_{n}^{A}$ is the normal contact stiffness of disks A; $\;k_{n}^{B}$ is the normal contact stiffness of disks B; $k_{S}^{A}$ is the tangential contact stiffness of disks A; and $k_{s}^{B}$ is the tangential contact stiffness of disks B.

The calibration method was to establish initial microscopic parameters for virtual penetration experiments, comparing the results of laboratory and numerical experiments based on these parameters. The microscopic parameters were adjusted incrementally until the simulation results approximated true value. Optimal parameters values for aggregates particles were determined through iterative adjustment based on the variation pattern of microscopic parameters, as shown in Table 4.

This study evaluates the degree of conformity between the numerical and laboratory results by using Mean Absolute Error (MAE), Root Mean Square Error (RMSE), and the Coefficient of Determination (R^2^). The evaluation indicator can be calculated as:

(3) $$\mathrm{MAE}=\frac{1}{n}\overset{n}{\underset{i=1}{\sum }}\left|y_{i}-{\hat{y}}_{i}\right|$$

(4) $$\mathrm{RMSE}=\sqrt{\frac{1}{n}\overset{n}{\underset{i=1}{\sum }}{{(y}_{i}-{\hat{y}}_{i})}^{2}}$$

(5) $$R^{2}=1-\frac{\sum_{i=1}^{n}{{(y}_{i}-{\hat{y}}_{i})}^{2}}{\sum_{i=1}^{n}{{(y}_{i}-{\bar{y}}_{i})}^{2}}$$

where $y_{i}$ is the actual value; ${\hat{y}}_{i}$ is the predicted value; ${\bar{y}}_{i}$ is the average of the actual values; and n is the total number of samples in the dataset.

Among these metrics, a smaller Root Mean Square Error (RMSE) value indicates greater stability in the simulation results. Mean Absolute Error (MAE) reflects the average magnitude of errors without considering their direction, and its value ranges from 0 to infinity. As the Mean Absolute Error (MAE) approaches 0, the accuracy of the simulation improves. The Coefficient of Determination (R^2^), ranges from 0 to 1, with higher values indicating a better agreement between the simulated and experimental values.

During compaction of graded aggregates, the aggregates were subjected to constant impact action. The distances between the aggregates were continuously decreasing. The aggregates deformation and contact force are constantly increasing. However, the contact force between particles can be different. Significant deformation and substantial contact forces are exhibited by certain particles, which bear greater external loads and gravitational forces, thus forming strong force chains. Conversely, other particles are dispersed within the interstices of larger particles, bearing smaller external loads and gravitational forces, thereby forming weak force chains. The strong and weak force chains collectively constitute the force chain network of the pavement subbase. These force chain networks permeate the entire road surface nonuniformly and adapt in response to variations in external loads.

The coordination number, which is a crucial metric in discrete element simulations, is employed to assess the adequacy of contact and the degree of compaction within a particle system. The average coordination number is defined as:

(6) $$C_{N}=\frac{\sum_{i}^{N_{p}}N_{P\cdot Ci}}{N_{p}}$$

where $C_{N}$ is the mean number of particles in contact with other particles; $N_{P\cdot Ci}$ is the number of contacts each particle has with others; $N_{p}$ is the total number of particles; and i is the identifier for each particle.

The analysis of the vibratory compaction process can be conducted from the perspectives of changes in force chains, coordination numbers, and contact force patterns. The vibratory compaction of the coupled model can be accurately represented by these changes.

### 2.3. Design Forms and Parameters of Optical Cable Structure

**Figure 4:**
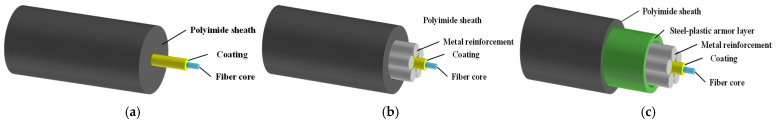
Structural design forms of different optical fiber cables: (**a**) Tight-buffered optical cable; (**b**) Optical fiber cable with GFRP composites; (**c**) Armored central tube optical fiber cable.

In order to investigate the effects of structural design forms and sizes on optical cable under the vibration compaction of pavement subbase, three representative optical cable structural design forms from Shandong Pacific Optical Fiber and Cable Co., Ltd. (Liaocheng, China) were employed, as illustrated in Figure 4. They were tight-buffered optical fiber cable, optical fiber cable with glass-fiber-reinforced polymer (GFRP) composites, and armored central tube optical fiber cable. As shown in Figure 4a, the tight-buffered optical fiber cable has an outer diameter of 9.0 mm, and the outer protective sheath had a thickness of 7.5 mm. The cable comprised an external polymer protective sheath and a fiber core. Figure 4b presents the optical fiber cable with GFRP composites, which had an outer diameter of 9.0 mm and an outer protective sheath thickness of 1.6 mm. GFRP components were adopted as reinforcing elements so that tensile resistance and structural stability could be improved. Figure 4c shows the armored central tube optical fiber cable, which had an outer diameter of 9.0 mm, an armor layer thickness of 0.5 mm, and an outer protective sheath thickness of 1.6 mm. The central-tube structure and the armored layer could improve resistance to external impact, abrasion, and compressive loading.

During the construction of an impact roller compactor, the optical cable in the subgrade is subjected to forces from all directions within the soil. Due to the length of each section of the cable is much larger than the outer diameter of the cross-section, the compression in the length direction is not considered. The simplification of the cross-section shape has little effect on the overall force on the cable. For the convenience of calculation, the cross-section of the cable is simplified to a square, the square side length is equal to the original cross-section of the circular outer diameter of the length [33].

The three-dimensional orientations of the optical cable are defined as follows. The *X*-direction runs along the length of the fiber-optic cable, the *Y*-direction is perpendicular to the fiber-optic cable and lies within the plane of the road panel, and the *Z*-direction is oriented vertically. To ensure that the optical cable could survive the pavement construction process and maintain coordinated strain transmission with the surrounding base materials, specific constraints were established. The most unfavorable stresses affecting the cable occur in the three orientations: tensile stress in the *X*-direction, and compressive stress in the *Y-* and *Z-direction*. The cable needs to meet the following stresses: (1) the tensile stress in the *X*-direction is less than the short-term allowable tensile stress; (2) the compressive stress in the *Y* or *Z* axes is less than the short-term allowable compressive stress; (3) the shear stress in the *Y* or *Z* axes is less than the allowable shear stress due to interface bonding; (4) the bending radius should exceed the minimum bending radius.

According to the Chinese standard T/CI 460-2024 [34], the physical properties of distributed optical fiber sensors were required to comply with the criteria listed in Table 5.

The tangential strain along the curved cable can be calculated as:

(7) $$\varepsilon_{\theta }=\ln\left|1+\frac{1}{4R}\left[D-2R-2t_{0}+\sqrt{{\left(2R+D-2t_{0}\right)}^{2}+16Rt_{0}}\right]\right|$$

where $t_{0}$ is the original wall thickness of the cable; *R* is the bending neutral layer radius of the cable; and *D* is the diameter of the distributed fiber-optic sensor.

The axial strain at a certain point on the optical cable can be calculated as:

(8) $$\varepsilon_{i}=\sqrt{1+{\left(\frac{d_{i}}{l}\right)}^{2}}-1$$

where $\varepsilon_{i}$ is the strain of a point of the cable; $d_{i}$ is the relative vertical displacement of a point of the cable; and *l* is the sampling point spacing of the cable.

In this study, the tangential strain of the optical cable bending curve was approximated as the axial strain of the optical cable. The relative vertical displacement of a specific point along the optical cable can be calculated as:

(9) $$d_{i}\approx l\cdot \sqrt{2\cdot \ln\left|1+\frac{1}{4R}\left[D-2R-2t_{0}+\sqrt{{\left(2R+D-2t_{0}\right)}^{2}+16Rt_{0}}\right]\right|}$$

Based on the data presented in Table 5, based on the cross-sectional dimensions and the loading mode of the optical cable, the short-term allowable tensile and compressive forces specified in the standard were divided by the cross-sectional area of the cable to be converted into equivalent allowable tensile and compressive stresses. The equivalent allowable tensile stress were determined to be 8.8 MPa, and the maximum allowable compressive stress were determined to be 6.4 MPa. To avoid the excessive local curvature caused by vibratory compaction, the maximum allowable displacement of the optical cable was calculated to be 18.2 mm by substituting the minimum bending radius into Equation (9).

## 3. Results and Discussion

### 3.1. Micro Index Evolution of Vibration Compaction Process

**Figure 5:**
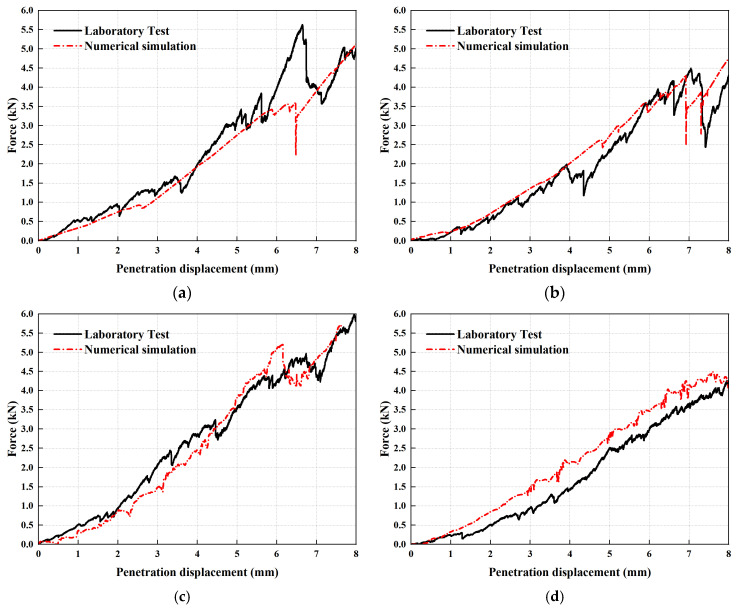
Comparison of force–displacement curves between the laboratory standard penetration test and the numerical simulation: (**a**) 13.2 mm aggregates; (**b**) 9.5 mm aggregates; (**c**) 4.75 mm aggregates; (**d**) 2.36 mm aggregates.

To assess agreement between the numerical simulations and laboratory measurements, we performed a statistical analysis. Table 6 compares the simulation and experimental results for the standard penetration test, and Figure 5 presents the comparison of force–displacement curves between the laboratory standard penetration test and the numerical simulation. The results presented in Figure 5 demonstrate that the simulated penetration curves for each aggregate size group were generally consistent with the corresponding laboratory curves, and the numerical curves exhibited a similar overall trend across aggregate size groups. While the overall trends of the numerical simulation curves are generally aligned, discrepancies in both the positions and amplitudes of fluctuations are observed in the ascending phase of the curves.

As shown in Table 6, the Coefficient of Determination (R^2^) for the 13.2 mm aggregates was only 0.76, which was lower than those of the other groups. By contrast, the values of R^2^ remaining three aggregate size groups maintained greater than 0.90. Across all aggregate size groups, the MAE ranged from 0.30 to 0.62, and the RMSE ranged from 0.36 to 1.00. For the 13.2 mm aggregates, the MAE was 0.62 and the RMSE was 1.00. The error metrics in 13.2 mm aggregates were comparatively large within all of the aggregate size groups. The larger error in 13.2 mm aggregates could be attributed to greater variability between samples, which is typically associated with coarser particle combinations. With fewer particles participating in load transfer, rearrangements in local force chains can cause significant and intermittent fluctuations in the force–displacement response. However, these minor deviations do not compromise the overall experimental accuracy. The numerical model can well represent the overall stress–strain trend during the penetration process of particles in the laboratory tests.

**Figure 6:**
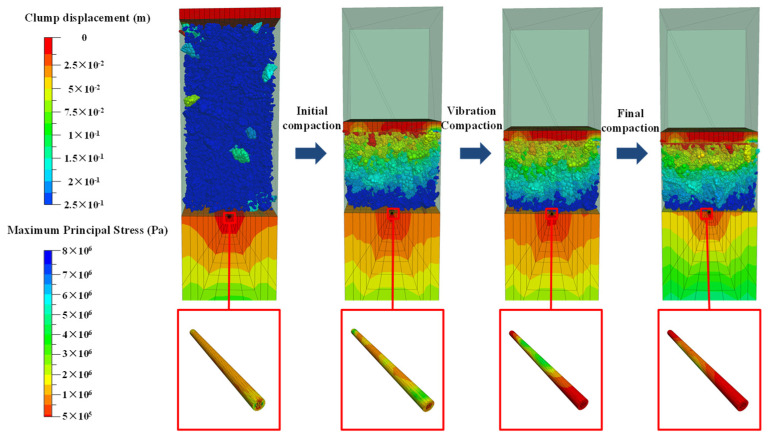
Vibration and compaction of graded aggregates particles and optical cable stress cloud diagram.

Figure 6 presents the cloud diagram of the stress variation in graded aggregates particles and optical cable in the process of initial compaction, vibration compaction and final compaction. During vibratory compaction, it is observed from the stress cloud diagram that the particles collectively migrate downward in the direction of the applied force. Movement initiation by larger particles leads to subsequent collisions and compression of smaller particles, thereby causing their motion. Under cyclic excitation, unstable contacts were initially disrupted, which caused the particles to rotate, slide, and redeposit. It transitioned into a more compact configuration. Migration of particles occurs from regions of higher stress to regions of lower stress, with instantaneous particle velocities increasing proportionally to larger amplitudes. The maximum stress of the optical cable is concentrated at the midpoint and decreases progressively towards both ends. The phenomenon occurred because the midpoint of the optical cable was subjected to the combined action of multiple particles and located farthest from the stress release boundaries at both ends, which facilitated the formation of stress concentrations.

**Figure 7:**
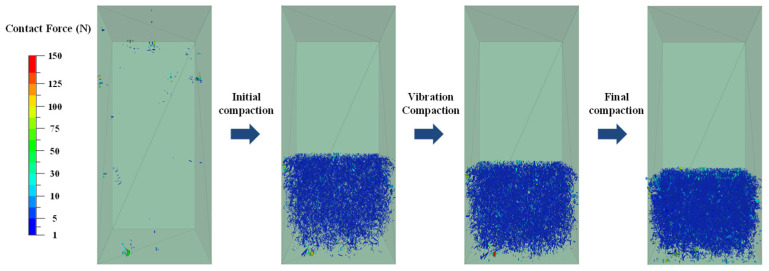
Variation in force chain of vibrational compacted aggregates.

Figure 7 shows the change in the vibration compaction aggregates particle force chain. The diagram shows weaker contact networks in blue and stronger ones in green. At the beginning of the compaction, there were only a few weak contact networks, and the contact force chain was thin. As compaction depth increases, weak contact networks expand significantly, while strong contact networks show gradual growth. It can be observed that the weak contact network provided geometric support for the rearrangement of particles, whereas the strong contact network constituted the primary load-bearing framework. The proliferation and thickening of stronger force chains indicate a progressive tightening of particle contacts, leading to an increase in interlocking forces and stabilization of the structure.

**Figure 8:**
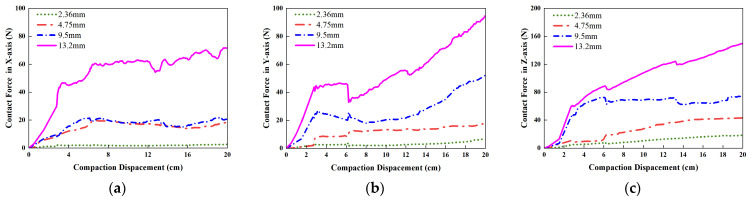
Variation in contact force in different particle sizes during vibratory compaction: (**a**) contact force in *X*-direction; (**b**) contact force in *Y*-direction; (**c**) contact force in *Z*-direction.

Figure 8 depicts the temporal variation in contact forces between four gradations of aggregates. The contact force in the *X*- and *Z*-direction of 13.2 mm aggregates increases significantly with the increase in compaction depth, followed by the *Y*-direction. The maximum contact force in the *X*-direction of 13.2 mm aggregates is approximately 100 N, and the maximum contact force in the *Y*-direction was about 70 N, the maximum contact force in the *Z*-direction exceeds 150 N. In contrast, the contact forces of the other three gradation do not exceed 50 N. It illustrates that the contact forces in the corresponding direction diminish progressively with decreasing particle diameter. The contact forces between large particles are considerably higher than that between small particles, which is the main resistance of the external load.

**Figure 9:**
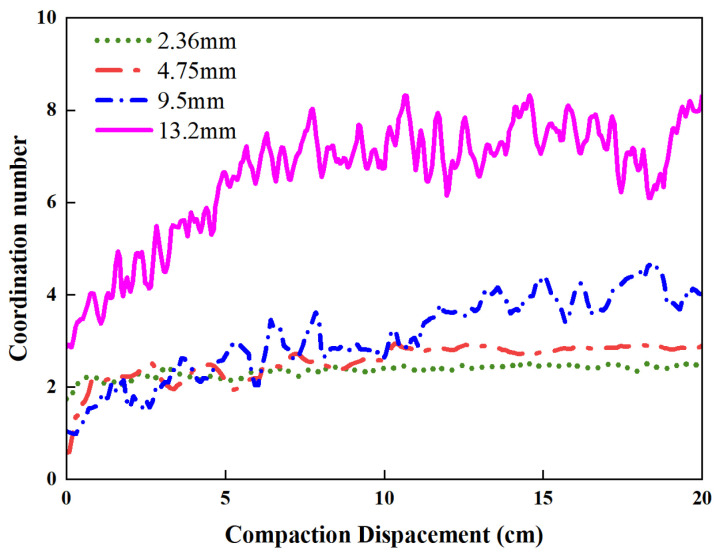
Variation in coordination number in different particle sizes during vibratory compaction.

Figure 9 shows the evolution law of the coordination number of different particle sizes during the vibrational compaction process. Similar to the aforementioned contact force variation, the aggregate coordination number decreases gradually with the decrease in particle diameter. In the four gradations of aggregates, the 13.2 mm aggregates has the most significant change, followed by 9.5 mm, 4.75 mm and 2.36 mm aggregates. The final coordination number of total model is about 8 and always slowly increases with compaction. The curves of the average coordination number and the contact force exhibited a consistent trend. The coordination number of the coarse aggregate particles increased significantly to yield larger values. It indicated that the coarse particles served as the primary load-bearing components under external vibratory loading.

In conclusion, during the numerical simulation of vibrational compaction, the contact force and coordination number of aggregates particles are increasing. The coordination number and contact force in the corresponding direction are gradually decreasing with the decrease in particle diameter. Thus, the simulated compaction process is verified effectively and can be simulated further by embedding optical fiber sensors in the virtual model.

### 3.2. Effects of Structure Design Forms on Optical Cable

**Figure 10:**
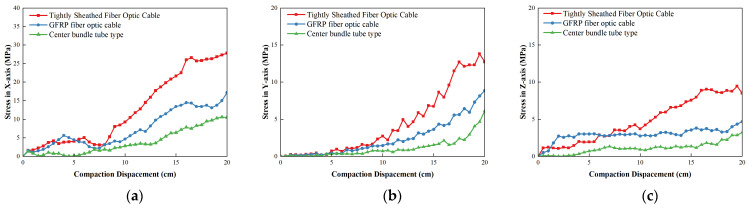
Variation in optical cable stress in different structure forms: (**a**) stress in *X*-direction; (**b**) stress in *Y*-direction; (**c**) stress in *Z*-direction.

Figure 10 shows the stress change in a tight sheath of unreinforced element cable. The GFRP reinforced element cable and central bundle tube armored cable increase the compaction depth during the compaction process which gradually increases triaxial stress of the cable. The maximum stress of the cable in the *X*-direction is 27.8 MPa, the maximum stress in the *Y*-direction is 13.8 MPa and the maximum stress in the *Z*-direction is 9.4 MPa. Among them, the maximum stress in the *Y*-direction was greater than the maximum allowable compressive stress 6.4 MPa, which exceeds the critical state. It indicated that the optical cable without strengthening elements of the tight sheath cannot withstand the repeated impact load during the vibration rolling of the road base which needs to be strengthened in the form of the optical cable structure. It could be attributed to the limited axial stiffness and radial load bearing capacity of the tight-buffered optical cable. Under the external loads, forces could be more directly transmitted to the core area of the cable. Consequently, the direct transmission was more likely to induce stress concentrations and irreversible signal attenuation.

After increasing the GFRP strengthening element the triaxial stress of the cable decreases significantly. The maximum stress in the *X*-direction of the GFRP strengthening element is reduced to 17.2 MPa, the maximum stress in the *Y*-direction is reduced to 8.9 MPa, the maximum stress in the *Z*-direction is reduced to 4.70 MPa and the triaxial stress meets the allowable stress conditions. Compared with the cable, the increase in the GFRP reinforcement element cable reduced the maximum stress in the *X*-direction of the cable by 38.3%, the maximum stress in the *Y*-direction by 35.7% and the maximum stress in the *Z*-direction by 50.2%. The structural stiffness was enhanced by the inclusion of GFRP reinforcing elements. The GFRP elements exerted a specific restrictive effect on the axial tension and bending deformation of the optical cable. Therefore, the peak values of the three-dimensional stress could be significantly reduced.

After adding the armored layer on the basis of GFRP strengthening element the maximum stress in the *X*-direction of the central bundle tube armored cable is reduced to 10.6 MPa, the maximum stress in the *Y*-direction is reduced to 6.2 MPa, the maximum stress in the *Z*-direction was reduced to 3.25 MPa and the triaxial stress meets the allowable stress conditions. Compared with GFRP reinforced element fiber cable the increased armored layer of the central bundle tube armored fiber cable reduced the maximum stress in the *X*-direction of the cable by 38.2%, the maximum stress in the *Y*-direction by 30.6% and the maximum stress in the *Z*-direction by 30.9%. The reinforced element and armored layer significantly reduce the triaxial stress of the cable while also effectively improve the tensile and compression resistance of the cable. Furthermore, the radial restraint and contact buffering capacities of the optical cable were enhanced by the armor layer. It enabled contact with the surrounding aggregates over a broader circumferential range, which improved the load resistance capability of the optical cable.

**Figure 11:**
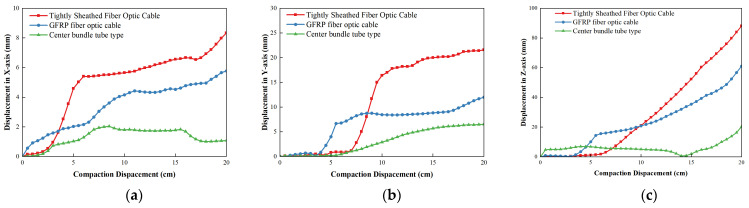
Displacement variation in optical cable of different structure forms: (**a**) displacement in *X*-direction; (**b**) displacement in *Y*-direction; (**c**) displacement in *Z*-direction.

Figure 11 shows the displacement change in the tight-buffered unreinforced element cable, GFRP reinforced element cable and central bundle tube armored cable. During the compaction process increase the compaction depth, the displacement in the *X*-direction of the tight-buffered unreinforced element cable increased rapidly and then gradually increases. The displacement in the *Y-* and *Z*-direction slowly increased and then rose gradually. The maximum displacement of the cable *X*-direction was 8.34 mm, the maximum displacement in the *Y*-direction was 21.6 mm and the maximum displacement in the *Z*-direction was 88.1 mm. The maximum displacement is within the allowable displacement range and far less than the allowable maximum displacement of 18.2 mm.

After adding the GFRP strengthening element, the displacement of the optical cable in the *X*-direction increases uniformly. The displacement of the *Y*-direction and the *Z*-direction increases rapidly at first and then slowly increases. For GFRP, the maximum displacement of the reinforcement element cable in the *X*-direction is 5.77 mm, the maximum displacement of the *Y*-direction is 12.0 mm, and the maximum displacement of the *Z*-direction is 61.0 mm. It is shown that GFRP strengthening element reduced the maximum *X* displacement by 30.8%, the maximum *Y* displacement by 44.4% and the maximum *Z* displacement by 30.8%.

After the cable was covered with armored layer, the displacement in the *X*-direction and displacement in the *Z*-direction slowly increased at a low level and the displacement in the *Y*-direction gradually and consistently increased. The maximum displacement of the central bundle tube armored cable in the *X*-direction decreased to 2.04 mm, the maximum displacement in the *Y*-direction was 6.54 mm and the maximum displacement of the *Z*-direction was 20.3 mm. As shown, the armored layer reduced the maximum displacement in the *X*-direction by 64.6%, the maximum displacement in the *Y*-direction by 45.5% and the maximum displacement in the *Z*-direction by 66.7%.

It is concluded that the reinforced element and armored layer significantly reduce the triaxial stress and triaxial displacement of the optical cable. Among them, the reinforced element improves the tensile and compression resistance of the optical cable more significantly so that it can withstand more tension and pressure in the use process. Moreover, both the displacement of the optical cable caused by external load and the spatial line error were reduced, and the stability along and reliability of the optical cable were improved.

### 3.3. Effects of Structural Parameters on Optical Cable

From Section 3.2, the reinforced element and armor layer can effectively improve the tensile and compression resistance of optical cable and reduce the spatial line error of optical cable. Among three optical cable structures, the mechanical performance of central bundle type armored optical cable was the best. Therefore, the central bundle tube armored optical cable structure was adopted further to investigate the effects of structure size on optical cable’s mechanical performance.

**Figure 12:**
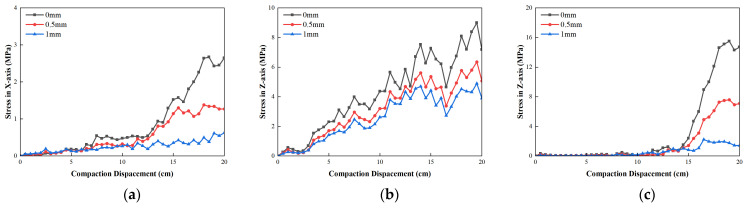
Stress variation in central bundle tube armored optical cable with different outer sheath thickness: (**a**) stress in *X*-direction; (**b**) stress in *Y*-direction; (**c**) stress in *Z*-direction.

Figure 12 shows the stress variation in a central bundle tube type armored optical cable with different outer sheath thickness. As shown in Figure 12, as the thickness of the outer sheath increases, the average compressive stress in the *X*-direction decreased, while the average compressive stress in the *Z*-direction increased and the average compressive stress in the *Y*-direction firstly increased and then decreased. The average *Y*-direction compressive stress with outer sheath thickness of 10 mm was the largest. After the outer sheath thickness increases from 9 mm to 11 mm, the maximum stress in the *X*-direction decreased from 4.63 MPa to 0.707 MPa, while the maximum *Y*-direction decreased from 9.68 MPa to 4.49 MPa and the maximum *Z*-direction decreased from 4.046 MPa to 1.55 MPa. The triaxial stress was within the allowable stress range. It shows that the outer sheath can improve the tensile ability of optical cable and has no obvious positive impact on the improvement of the compression capacity of optical cable under the compaction process. The outer sheath primarily functioned to provide geometric protection and localized buffering. An increase in the thickness of the outer sheath was found to reduce the axial stress by expanding its cross-sectional area. However, the resistance to concentrated radial compression was not significantly enhanced.

**Figure 13:**
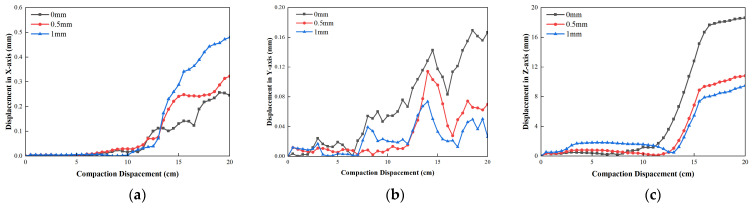
Displacement variation in different outer sheath thickness: (**a**) displacement in *X*-direction; (**b**) displacement in *Y*-direction; (**c**) displacement in *Z*-direction.

Figure 13 shows the displacement change in central bundle tube armored optical cable with different outer sheath thicknesses. As shown in Figure 13, it is noted that as the thickness of the outer sheath increases, the average displacement in the *X*-direction firstly increases and then decreases, whereas the average displacement in the *X*-direction of the thickness of 10 mm is the largest. The mean displacement in the *Y*-direction and *Z*-direction all decrease. And the dis-placement in the *Z*-direction almost never decreases after the outer sheath thickness exceeds 10 mm. When the sheath thickness increases from 9 mm to 11 mm, the maximum displacement in the *X*-direction is reduced from 0.3 mm to 0.5 mm, while the maximum displacement in the *Y*-direction is reduced from 1.7 mm to 0.74 mm and the maximum displacement in the *Z*-direction is reduced from 18.6 mm to 9.5 mm. It shows that with the increase in the outer sheath thickness, there is no obvious positive effect on the axial line position error of the optical cable under the compaction process. The thickening of the outer sheath merely improved the capability to control radial deformation. Once its buffering effect reached a specific threshold, further thickening did not significantly enhance the overall load-carrying capacity of the optical cable.

**Figure 14:**
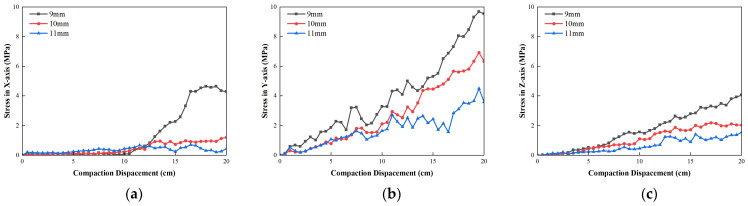
Stress variation in different armor layers: (**a**) stress in *X*-direction; (**b**) stress in *Y*-direction; (**c**) stress in *Z*-direction.

Figure 14 shows the stress change in different armored layers. As illustrated in Figure 14, the triaxial stress of the optical cable is reduced with the increase in armored layer thickness and the triaxial stress of the optical cable with different armored layer thicknesses is within the allowable stress range. As the increase in armored thickness, the maximum stress in the *X*-direction decreased from 15.5 MPa to 2.23 MPa, the maximum stress in the *Y*-direction decreased from 8.99 MPa to 4.896 MPa and the maximum stress in the *Z*-direction decreased from 2.67 MPa to 0.626 MPa. It shows that increasing the thickness of the armored layer can improve the tensile ability and compression ability of the optical cable effectively under the variation compaction. As the primary radial load-bearing component, when the thickness of the armor layer was increased, it directly improved the circumferential stiffness. It caused the contact loads to dissipate along a more extensive structural path. It indicated that increasing the thickness of the armor layer could enhance both the tensile and compressive resistances of the optical cable during compaction.

**Figure 15:**
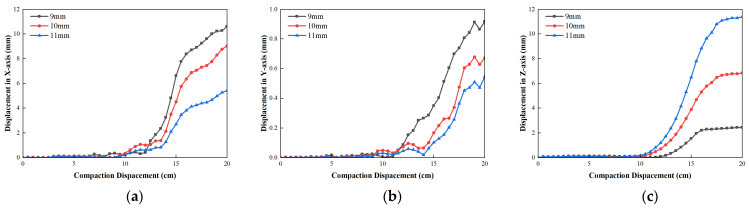
Displacement variation in different armor layers: (**a**) displacement in *X*-direction; (**b**) displacement in *Y*-direction; (**c**) displacement in *Z*-direction.

Figure 15 shows the displacement change in central bundle tube armored optical cable with different armored layer thickness. As shown in Figure 15, as the increase in the armored layer thickness, a change in the triaxial displacement of the optical cable is reduced. As the increase in armored thickness, the Z displacement is greatest and the maximum Z displacement is reduced from 2.45 mm to 11.4 mm, while the maximum X displacement is reduced from 10.61 mm to 5.41 mm and the maximum Y displacement is reduced from 0.92 mm to 0.55 mm. It shows that increasing the thickness of the armored layer can effectively reduce the radial and axial line position error of the optical cable under the compaction process. The increase in the thickness of the armor layer enhanced the impact resistance of the optical cable and its ability to coordinate deformation with the surrounding particulate medium.

## 4. Conclusions

To investigate the performance change in optical fiber sensor during pavement subbase construction, DEM-FDM combining the discrete element method with the finite difference method was utilized for dual-scale coupled numerical simulation. Considering the discrete characteristics of graded aggregates, the discrete element software package Particle Flow Program (PFC3D) was used to describe the graded aggregates and finite differential software Fast Lagrangian Analysis of Continuum (FLAC3D) to describe DFOS to establish the coupling model of distributed fiber sensor embedded in pavement structure. In order to represent the most unfavorable pavement construction conditions, the distributed fiber sensor was located on the top of the subgrade. Under vibratory compaction of the pavement subbase, the effects of cable structural design form and sizes on the mechanical performances and the spatial position error of optical cable were analyzed. The main conclusions are as follows:

(1) The dual-scale coupling model of optical cable and pavement is established and the vibration compaction process of the model is simulated. With the increase in compaction depth, the weak contact network increases significantly, the strong contact network rises slowly, the strong chain increases, the contact force and coordination number of aggregates particles are increasing and the final coordination number is about 8. With the decrease in particle diameter, the coordination number and contact force in the corresponding direction are gradually decreasing. In the four gradations of aggregates, the contact force of 13.2 mm increases significantly with the increase in compaction depth. The maximum contact force of 13.2 mm aggregates in the *X*-direction approaches 100 N, the maximum contact force in the *Y*-direction is about 70 N, and the maximum contact force in the *Z*-direction exceeds 150 N, which is the main resistance of the external load.
(2) The reinforced element and armor layer in the optical cable structure can effectively improve the tensile and pressure resistance performance of the optical cable, reducing the spatial line position error caused by road construction. The maximum stress in the *Y*-direction of the tight-buffered optical cable without reinforcement element exceeded the maximum permissible compressive stress. The tight-buffered optical cable is incapable of withstanding vibrational impact loads. After the addition of the GFRP strengthening element, the triaxial stress and displacement of the cable decreased significantly, and the maximum stress in the *X*-direction, *Y*-direction and *Z*-direction were reduced by 38.3%, 35.7% and 50.2%. The maximum displacement in the *X*-direction, *Y*-direction and *Z*-direction was reduced by 30.8%, 44.4% and 30.8%, meeting the allowable stress and displacement conditions. After adding the armored layer to the cable with GFRP strengthening elements, the maximum stress of the cable in the *X*-direction, *Y*-direction and *Z*-direction were reduced by 38.2%, 30.6% and 30.9%. The maximum displacement in the *X*-direction, *Y*-direction and *Z*-direction were reduced by 64.6%, 45.5% and 66.7%. After adding the GFRP strengthening element or armored layer, the triaxial stress and spatial line position error of the optical cable were reduced. It indicated that these reinforcing components could effectively enhance the short-term mechanical performance of the cable. From the perspective of engineering applications, although the construction survivability and mechanical protection capabilities of the sensing optical cable were improved by the GFRP reinforcements and armor structures, the long-term performance has not yet been verified. Therefore, the structural selection and design of the optical cable should be comprehensively determined by evaluating the mechanical protection capabilities, installation feasibility, and overall monitoring costs.
(3) The increase in outer sheath thickness can improve the tensile capacity of the cable and reduce the radial line position error of the cable, while it has no obvious positive impact on the improvement of the compression capacity of the cable and the axial line position error. With the increase in the outer sheath thickness, the average *X*-direction stress of the central bundle tube armored cable decreases, and the average *Z*-direction compressive stress increases. The average *Y*-direction compressive stress increases firstly and then decreases, and the average *Y*-direction compressive stress of the outer sheath thickness of 10 mm is the largest. The increase in the outer sheath thickness causes the average displacement of the optical cable in the *X*-direction to first increase and then decrease, and the average displacement of the thickness 10 mm in the *X*-direction was the largest. The mean Y displacement and the mean Z displacement decrease, and after the outer sheath thickness reaches 10 mm, the Z displacement almost never decreases. When outer sheath thickness increases from 9 mm to 11 mm, the maximum stress in the *X*-direction, maximum stress in the *Y*-direction and maximum stress in the *Z*-direction of the cable were reduced by 84.7%, 53.6% and 61.7%.
(4) The increase in the thickness of armored layer can improve the ability of optical cable to withstand tensile and compression, and effectively reduce the radial and axial line position error of optical cable. With the armored-layer thickness increasing from 0 to 1 mm, the maximum stress of the cable in the *X*-direction, *Y*-direction and *Z*-direction were reduced by 85.6%, 45.5% and 76.6%.
(5) The monitoring of pavement surface conditions constitutes a prolonged and complex endeavor. While traditional point-type sensors encounter significant difficulties in capturing continuous strain distributions under traffic loading and construction effects, DFOS systems are capable of continuously acquiring structural responses with high spatial resolution. Owing to the constraints of the existing experimental conditions, the complete compaction process involving embedded optical cables could not be verified through in situ measurements or controlled laboratory experiments. The simplified subgrade model utilized in this study failed to account for the nonlinear response and moisture-dependent behaviors of actual soils. Furthermore, the influence of the thermo-mechanical coupling effects of hot-mix asphalt (HMA) concrete during high-temperature paving on the survivability of the optical cables was not considered. In the future research, large-scale vibratory compaction tests incorporated with embedded DFOS alongside in situ monitoring could be conducted. The thermo-mechanical response mechanisms of DFOS under the high-temperature construction conditions of asphalt layers will be subsequently explored. Reliable support can be established for the life cycle performance evaluation of pavements based on DFOS by coupling the mechanical performance analysis of DFOS with long-term durability evaluations.

## Figures and Tables

**Table 1:** Parameters for the distributed fiber optical cable and pavement.

Layer	Materials	Density (kg/m^3^)	Size (mm)	Elastic Modulus (MPa)	Poisson Ratio
Optical fiber core	Fused quartz	2203	0.13	71,700	0.17
Coating	PBT	1400	0.5	5700	0.3
Metal reinforcement	GFRP	2000	1	17,200	0.29
Armored layer	Steel	7850	0.5	206,000	0.3
Outer sheath	Polyimide	961	1.6	1170	0.3
Subbase	Graded aggregates	2700	200	500	0.35
Subgrade	Clay	1800	400	60	0.4

**Table 2:** Gradation for aggregate skeletons.

Sieving Size (mm)	13.2	9.5	4.75	2.36
Passing ratio (%)	95	77	59	38

**Table 3:** Parameters of the vibratory roller [31].

Parameter	Unit	Value
Drum mass	kg	13,000
Excited force	kN	405
Excitation frequency	Hz	32
Nominal amplitude	mm	0.95
Drum width	mm	2170
Drum diameter	mm	1600

**Table 4:** Microscopic parameters of intergranular contact.

Parameter	Unit	Clump	Coupling Wall	Boundary Wall
Normal contact stiffness *k_n_*	Pa	5 × 10^6^	1 × 10^7^	1 × 10^10^
Shear contact stiffness *k*_s_	Pa	5 × 10^6^	5 × 10^6^	1 × 10^10^
Friction coefficient	-	0.5	-	-
Density	kg/m^3^	2700	-	-

**Table 5:** Physical performance of distributed fiber-optic sensors.

Function		Metric
Allowable Tensile Force	Long-term	2000 N
	Short-term	3000 N
Allowable Compressive Force	Long-term	2000 N/10 cm
	Short-term	3000 N/10 cm
Minimum Bending Radius	Static	20 D *
	Dynamic	30 D *

* D is the diameter of the distributed fiber-optic sensor.

**Table 6:** Comparison of laboratory and simulation results of the standard penetration test.

Evaluation Indicator	13.2 mm	9.5 mm	4.75 mm	2.36 mm
MAE	0.62	0.30	0.46	0.38
RMSE	1.00	0.36	0.70	0.44
R^2^	0.76	0.96	0.92	0.93

## Data Availability

The original contributions presented in the study are included in the article. Further inquiries can be directed to the corresponding author.
